# Investigating Structural and Surface Modifications in Ion-Implanted 4H-SiC for Enhanced Dopant Distribution Analysis in Power Semiconductors

**DOI:** 10.3390/ma17235734

**Published:** 2024-11-23

**Authors:** Taehun Jang, Mirang Byeon, Minji Kang, Sang-Gil Lee, Ji Hyun Lee, Sang-Geul Lee, Won Ja Min, Tae Eun Hong

**Affiliations:** 1 Busan Center, Korea Basic Science Institute, Busan 46742, Republic of Korea; cry457@kbsi.re.kr (T.J.); bmr12@kbsi.re.kr (M.B.); mjkang@kbsi.re.kr (M.K.); 2 Department of Materials Engineering, Pusan National University, Busan 52828, Republic of Korea; 3 Research Center for Materials Analysis, Korea Basic Science Institute, Daejeon 34133, Republic of Korea; lsg0418@kbsi.re.kr (S.-G.L.); jih5838@kbsi.re.kr (J.H.L.); 4 Daegu Center, Korea Basic Science Institute, Daegu 41566, Republic of Korea; sagelee@kbsi.re.kr; 5 Business Development Team, HB Solution, Daejeon 34028, Republic of Korea; wj.min@hb-solution.co.kr

**Keywords:** ion implantation, silicon carbide, power semiconductor, secondary ion mass spectrometry, quantitative analysis, surface morphology

## Abstract

This study aims to develop a reference material that enables precise management of dopant distribution in power semiconductors. We thoroughly investigate the structural and surface properties of 4H-silicon carbide (4H-SiC) single crystals implanted without annealing using aluminum (Al) and phosphorus (P) ions. Ion-implanted 4H-SiC was thoroughly evaluated using advanced techniques, including X-ray diffraction (XRD), field emission transmission electron microscopy (FE-TEM), atomic force microscopy (AFM), time of flight medium energy ion scattering (ToF-MEIS), and secondary ion mass spectrometry (SIMS). The evaluated results indicate that, without post-annealing, ion-implanted 4H-SiC can serve as an effective reference material for the precise control of trace elements and the quantitative monitoring of dopant distribution in power semiconductor applications.

## 1. Introduction

Silicon carbide (SiC) is now an indispensable core material in the power semiconductor industry due to its excellent properties such as high thermal conductivity, wide bandgap, and chemical inertness. The superior properties of SiC, which have been studied for decades, include a larger bandgap along with higher thermal conductivity, breakdown field and carrier saturation velocity [1,2,3]. Compared to 3C- and 6H-, 4H-SiC is distinguished by superior physical properties with respect to bandgap, electron, and hole mobility [2,3,4]. In particular, 4H-SiC has attracted great attention for power electronics applications because semiconductor devices based on 4H-SiC can operate at high voltages, high temperatures, and high switching speeds [3,5,6]. Due to its advantageous properties, 4H-SiC is a highly suitable material for applications in extreme environments. However, paradoxically, the very characteristics that enable 4H-SiC to endure extreme environments also make conventional thermal diffusion processes challenging. As a result, selective doping in 4H-SiC has primarily been accomplished via ion implantation [7,8]. This method has become indispensable for producing high-quality power semiconductor transistors, as it enables precise dopant control. Nevertheless, a comprehensive understanding of the structural changes and defect generations during ion implantation remains essential [9,10,11]. Moreover, continuous evaluation and monitoring of ion-implanted results are required to ensure the industrial application of 4H-SiC [12,13].

A representative analysis technique for accurately monitoring dopants and trace elements in power semiconductors is secondary ion mass spectrometry (SIMS). SIMS, which analyzes secondary ions generated by bombarding primary ions on the sample surface, is a mass spectrometry method that can be detected from several ppm to ppb and is suitable for monitoring power semiconductors whose electrical characteristics vary dramatically due to dopant injection. For stable SIMS monitoring, a constant ionization yield is required during SIMS analysis, and factors affecting the ionization yield, such as samples’ amorphization, surface roughness, lattice defects, and surface oxidization, should be controlled [14,15,16]. To manage these variables, a reference material must require an appropriate concentration peak (C_p_) and projected range (R_p_). Ion implantation is also a useful method for fabricating reference materials to form suitable C_p_ and R_p_. However, various reported studies have shown that ion implantation affects the surface and lattice properties of 4H-SiC [17,18,19]. For SIMS doping concentration analysis in power semiconductors, the surface roughness of 4H-SiC, used as a reference material, must be maintained below 1 nm to ensure a flat surface and a stable lattice structure [20,21,22]. Although numerous previous studies have been conducted, the majority primarily focused on the electrical activation of 4H-SiC dopants, the effects of annealing on improving surface properties, and conventional semiconductor materials such as Si and SiO_2_. [17,18,19,23,24].

In this study, we thoroughly investigated the structural and surface properties of ion-implanted 4H-SiC single crystal samples, prepared without post-annealing, to evaluate their potential as reference materials. The samples were implanted with two different ions (Al and P) and characterized using X-ray diffraction (XRD), field emission transmission electron microscopy (FE-TEM), atomic force microscopy (AFM), time of flight medium energy ion scattering (ToF-MEIS), and secondary ion mass spectrometry (SIMS).

## 2. Materials and Methods

### 2.1. Ion Implantation of 4H-SiC

Four-inch n-type 4H-SiC wafers, off-axis 4.0° toward the (1120) lattice plane, with a thickness of 350 µm and a nitrogen doping concentration of 1 × 10^19^ atoms/cm^−3^, were used. Al and P ions were used for p-type and n-type implantation, respectively. The ion implantation was conducted on the commercial 4-inch n-type 4H-SiC wafer with an energy of 150 keV at room temperature (RT), and post-implantation thermal treatment was not applied. Neither encapsulation nor external pressure sources were employed in the samples. To evaluate surface and lattice properties, 4H-SiC samples were precisely diced using a dicing saw, resulting in 10 mm × 10 mm pieces for each samples. The samples in this paper designated none, Al, and P in 4H-SiC were assigned to denote S1, S2, and S3, respectively. Detailed experimental conditions and identification parameters for the identification of the sliced 4H-SiC single crystals are presented in Table 1.

### 2.2. Characterization of Surface and Structural Properties

#### 2.2.1. X-Ray Diffraction

The crystallographic and structural properties of the ion-implanted 4H-SiC were determined by high-resolution X-ray diffraction (Empyrean, Malvern Panalytical, Malvern, UK) at the Korea Basic Science Institute (KBSI; Daegu, Republic of Korea). Cu-Kα_1_ radiation (λ = 1.541 Å) was used at an accelerating voltage of 40 kV and current of 30 mA.

#### 2.2.2. Transmittance Electron Microscope

To investigate their crystal structure, field emission transmission electron microscopy (field emission−TEM) images of the 4H-SiC single crystal samples were taken after the ion implantation process. The TEM images and selected area electron diffraction (SAED) patterns of the ion-implanted 4H-SiC single crystal samples were taken with a ZrO/W emitter (JEM 2100F, JEOL, Akishima, Japan) transmission electron microscope, operating at 200 kV.

#### 2.2.3. Atomic Force Microscope

The surface roughness of the ion-implanted 4H-SiC single crystals was performed at a non-contact mode using an atomic force microscope (AFM; NanoWizard II, Bruker, Berlin, Germany) at the Korea Basic Science Institute (KBSI; Busan, Republic of Korea). All the measurements were obtained under controlled room temperature. The scanning area was 10 μm × 10 μm and scan rate was set at 1.5 Hz. The Bruker NanoWizard software (SPM Software 3.3a) was used for image processing and interpretation.

#### 2.2.4. Time of Flight Medium Energy Ion Spectroscopy

To identify differences in surface defect characteristics of the ion-implanted 4H-SiC crystal samples, the ion-implanted 4H-SiC samples were measured by a time-of-flight medium energy ion scattering with sub-nanometer depth resolution. Time of flight medium energy ion spectroscopy (ToF-MEIS; MEIS K-120, HB Solution, Daejeon, Republic of Korea) spectra were obtained using a helium ion (He^+^) beam with an energy of 100 keV. The ion beam pulse width was 350 ps, and the resolution was approximately 7 × 10^−3^ (ΔE/E). The typical beam size for the sample was 500 μm^2^, and the ion beam was rastered to 5 mm × 5 mm, limiting the ion dose per unit area of the sample to 1.5 × 10^13^ ions/cm^2^. The detector solid angle was 76.6 mSr. To evaluate the crystallinity of the sample, channeling spectra were acquired under double alignment conditions with 49° incident angle and 135° scattering angle, respectively. Since the sample is a crystal 4° off-axis, the 49° incident angle and the 135° scattering angle correspond to the c-plane of 4H-SiC (0001) direction. Each data is collected for 600 nC per spectrum, which provides a typical error less than 5% in crystallinity in the extracted data. The ToF-MEIS spectra were analyzed using MEISis2.0 provided by the manufacturer, and Anderson screening, Anderson–Ziegler stationary cross-section, and Yang straggling were applied in analysis.

#### 2.2.5. Magnetic Sector Secondary Ion Mass Spectrometry

To quantitatively monitor the elemental depth distribution in the implanted 4H-SiC single crystal, magnetic sector secondary-ion mass spectrometry (magnetic sector−SIMS; IMS-7f, Cameca, Gennevilliers, France) at the Korea Basic Science Institute (KBSI; Busan, Republic of Korea) was used. For the magnetic sector SIMS measurements, the ^31^P^−^ secondary ions were acquired at a high mass resolution using a cesium ion (^133^Cs^+^, 15 keV), while the ^27^Al^+^ secondary ions were acquired using oxygen ions (^16^O_2_^+^, 7.5 keV). The primary ion was rastered over an area of 150 × 150 μm^2^, and the secondary ion signal was recorded from the central part of this area (~30 μm diameter).

#### 2.2.6. Stylus Profiler

After the magnetic sector SIMS depth profiling was completed, the depth of the formed crater was measured using a stylus profiler (P-7, KLA Tencor, Milpitas, CA, USA). The sputter depth of the 4H-SiC sample was measured at the Korea Basic Science Institute (KBSI; Busan, Republic of Korea) using a stylus, which can directly measure the crater depth by using a diamond stylus that contacts the sample laterally and moves vertically. This allowed for the conversion of sputtering time into analysis depth.

## 3. Results and Discussion

**Figure 1:**
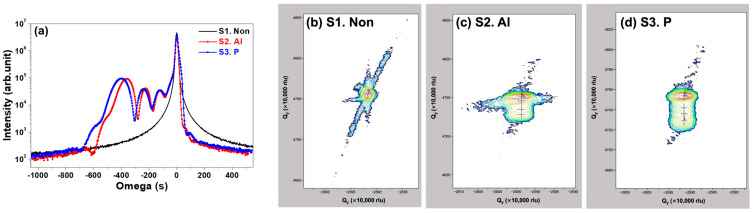
(**a**) XRD omega rocking curves of 4H-SiC single crystals with three different ion-implanted samples and RSM images obtained from three different ion-implanted samples: (**b**) none, (**c**) Al, and (**d**) P ion.

To identify the defects in the ion-implanted 4H-SiC single crystals, we measured XRD omega rocking curves on three different ion-implanted samples: S1, S2, and S3. As shown in Figure 1a, the non-implanted sample S1 exhibits a high-intensity peak corresponding to the (0004) of 4H-SiC (JCPDS 01-073-1664). The highest peak observed in all three samples corresponds to the 4H-SiC (0004) reflection. The full width half maximum (FWHM) of the (0004) peak is 12.9, 15.5, and 14.7 for S1, S2, and S3, respectively. The increased FWHM observed in the ion-implanted samples (S2 and S3), compared to the non-implanted sample (S1), indicate the effect of the ion implantation process on crystallinity. Nevertheless, the small difference in FWHM shows that the d-spacing of the 4H-SiC single crystal is confirmed to remain stable following ion implantation. As seen in Figure 1a, the ion-implanted samples (S2 and S3) show the (0004) peak of 4H-SiC and three distinct peaks at lower angles relative to the substrate peak. The two small peaks close to the substrate peak indicate that ions were implanted in the surface region of the 4H-SiC single crystal, which is known to cause typical strain due to ion implantation [25,26]. The second prominent peak observed near −400 arcseconds in S2 and S3 is believed to correspond to a layer of Al and P ions formed through ion implantation. To analyze the strain variation more specifically, we also conducted RSM measurements of S1, S2, and S3.

The RSM measurement of the asymmetrical (106) reflection obtained from the 4H-SiC with three different ion-implanted samples, S1, S2, and S3, are shown in Figure 1b–d. As with the non-implanted 4H-SiC in Figure 1b, the region of the strongest intensity, which is the 4H-SiC substrate, is also shown in Figure 1c,d. What makes S2 and S3 different from S1 is that several different peak intensities appear below the highest intensity. Examining the magnified RSM image reveals the presence of three fringes, which resemble the findings obtained from rocking curve measurement results [26,27]. The ion-implanted SiC structural layer exhibits a vertical strain (ΔQ_z_) comparable to that of the SiC substrate in the RSM results, while the horizontal strain (ΔQ_x_) is observed to be zero, indicating full strain accommodation relative to the substrate [28,29]. This result confirms that only vertical strain was induced in the crystal structure, despite the vertical incidence of ion implantation on the 4° off-axis SiC substrate sample. Rocking curve measurements and RSM analysis confirmed that, despite slight deformation in the ion-implanted 4H-SiC, overall structural distortion is minimal, with crystallinity remaining consistently uniform, particularly along the a-plane.

**Figure 2:**
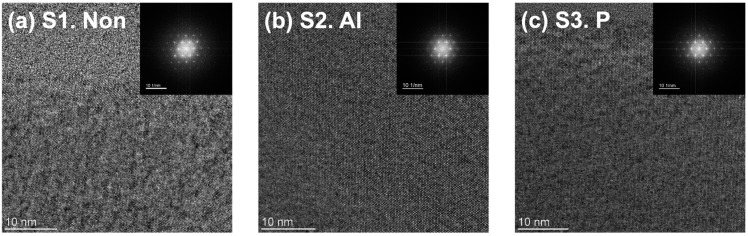
Planar TEM images and SAED patterns of 4H-SiC single crystal for three distinct ion-implanted samples: (**a**) none, (**b**) Al, and (**c**) P ion implantation.

The microstructure of the implanted 4H-SiC single crystals was characterized by high-resolution TEM measurements. Figure 2a–c present the planar TEM images and SAED patterns of the 4H-SiC single crystal across three different ion implantation conditions: non-, Al-, and P-implanted 4H-SiC. For all cases, the planar TEM images were captured on 4H-SiC matrices containing non-, Al-, and P-implanted specimens. As a result of planar TEM measurements, no discernible defects or vacancies were observed within the periodic lattice structure. Additionally, all corresponding SAED patterns also reveal the stable status of the lattice structure. In other words, it can be seen that damage to the crystal lattice of the 4H-SiC surface was effectively controlled by maintaining the appropriate temperature during ion implantation.

**Figure 3:**
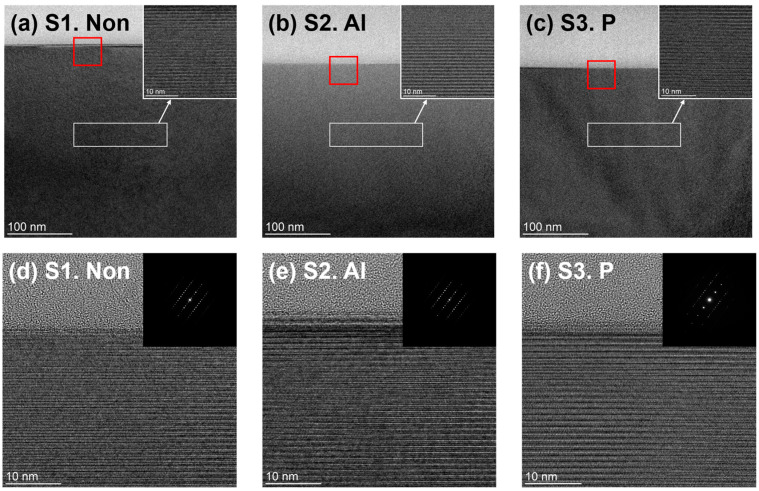
Cross-sectional TEM images and SAED patterns of 4H-SiC single crystals at low (**a**–**c**) and high (**d**–**f**) magnification for three ion-implanted samples, S1, S2, and S3. Red square areas in (**a**–**c**) are the areas measured by high-magnification TEM in (**d**–**f**).

Figure 3a–f show high- and low-magnification cross-sectional TEM images and SAED patterns of 4H-SiC single crystals using three different ion-implanted samples (S1, S2, and S3). Cross-sectional TEM imaging effectively reveals lattice defects and damage caused by ion implantation at both the surface and at specific depths (Projected range, R_p_) [30]. In the low-magnification cross-sectional TEM images presented in Figure 3a–c, no significant lattice defects or damage are observed, even at depths of approximately 250 nm, which exceeds R_p_, where ion implantation damage could be greatest [12,16]. Notably, high-magnification TEM analysis confirmed that there was no significant damage to the 4H-SiC lattice even in the white-boxed regions near R_p_ in Figure 2b,c, where the concentrations of Al and P ions were highest.

To specifically confirm the condition of the surface of samples, high-magnification cross-section TEM was measured near the surface, as shown in the red box of Figure 3a–c along with SAED patterns, and the results are shown in Figure 3d–f. Analogous to the planar TEM and low-magnification results, the near-surface cross-sectional TEM and SAED images exhibit a uniform crystal structure [30]. From the planar and cross-sectional TEM results, we confirmed that the ion-implanted and non-implanted samples were homogeneous structures without lattice defects or damage.

**Figure 4:**
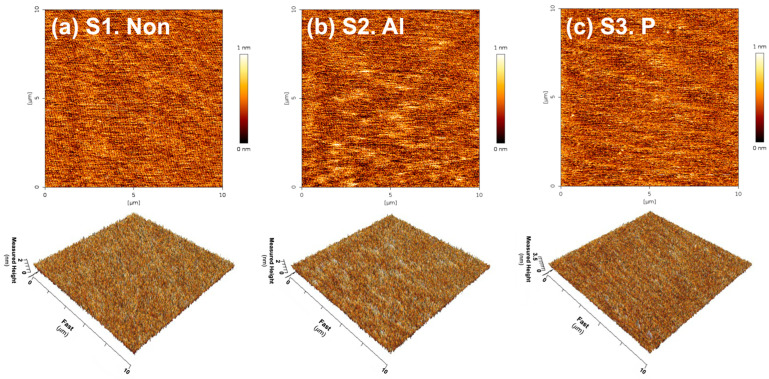
2D and 3D AFM images of a 4H-SiC single crystal under three different ion implantation conditions: (**a**) none, (**b**) Al ions, and (**c**) P ions (S1, S2, and S3).

Figure 4a–c present atomic force microscope (AFM) images of 4H-SiC single crystals subjected to three different implantation conditions: none, Al, and P ions (denoted as S1, S2, and S3). As illustrated in Figure 4a–c, the average surface roughness (R_a_) and the root mean square roughness (R_rms_) for samples S1, S2, and S3 were each determined to be below 0.20 nm and 0.25 nm, respectively. From these results, we confirmed that Al and P ions were implanted without change in the surface roughness of the three samples before and after implantation. Table 2 summarizes the detailed surface roughness of a 4H-SiC single crystal with respect to three different sample conditions.

**Figure 5:**
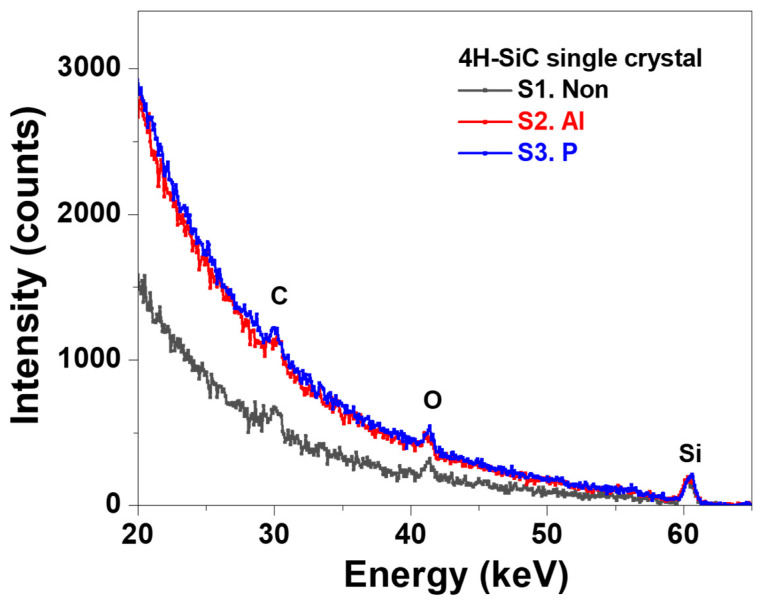
ToF-MEIS spectra of the ion-implanted 4H-SiC single crystal samples with three different conditions, namely none, Al, and P ion implantation (S1, S2, and S3).

ToF-MEIS is a useful method for identifying lattice damage by analyzing the channeling spectrum [31,32,33]. Figure 5 shows the channeling spectra of non-implanted 4H-SiC and Al and P ion-implanted 4H-SiC, S1, S2, and S3, respectively. When the ToF-MEIS ion beam is incident along the crystal direction, the scattered intensity increases due to dechannelling as the beam propagates deeper into the crystal [32]. Consequently, the scattered intensity from surface Si is observed to be near 61 keV, while the scattered intensity from Si at a depth of 100 nm appears at 20 keV. Apart from the O and C peaks on the surface, which occur between 59 keV and 20 keV in the crystal region, the spectrum shows a smooth increase in yield without distinct peaks. This characteristic dechannelling behavior in a single crystal confirms that the crystallinity remains unchanged. Based on the MEIS spectra, we observed that the crystallinity distribution is uniform from the surface to a depth of approximately 200 nm, allowing for crystallinity assessment using the minimum yield. The minimum yield is defined as the ratio of the minimum height in the channeling spectrum to the expected height of a random spectrum at the same energy [32,33]. Under the fixed experimental conditions of this measurement, the minimum yield is directly proportional to the crystallinity of the sample surface. The minimum yield was 0.01 for S1 and 0.02 for S2 and S3. These values indicate that the Al- and P-doped samples were slightly less crystalline than the undoped 4H-SiC but still maintained good crystallinity. This suggests that minimal change occurred due to ion implantation under the experimental conditions.

**Figure 6:**
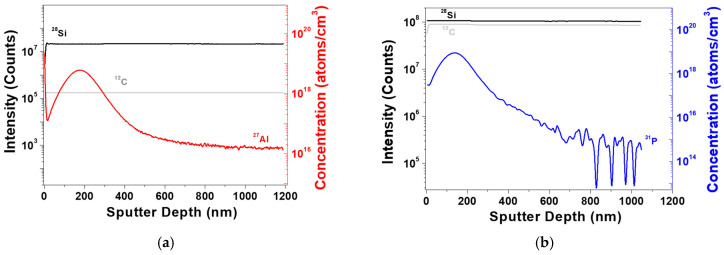
SIMS depth profiles of 4H-SiC single crystals with two different ion-implanted samples ((**a**) S2 and (**b**) S3).

Figure 6a,b show the SIMS depth profiles of 4H-SiC single crystal samples ion-implanted with Al and P. In the SIMS depth profiles for these two samples (S2 and S3), Al and P are observed to form a background at approximately 750 nm. The low intensity of ^12^C shown in Figure 6a is due to the low secondary ionization yield from the oxygen ion source used to enhance the sensitivity of the ^27^Al analysis [34]. It is evident that the P-implanted 4H-SiC samples exhibit a higher peak concentration compared to the Al-implanted samples. This results in a distinct crystallinity characteristic of the secondary peak layer in the XRD rocking curve and RSM results shown in Figure 1. Additionally, it was confirmed that the R_p_ for Al is greater than that for P, indicating that the secondary peak of the XRD rocking curve is closer to the substrate. To monitor the dopant quantitative distribution analysis, SIMS requires the R_p_, C_p_, and dose of reference materials for quantitative analysis. The results in Figure 6a,b display profiles suitable for use as reference materials [14,35,36]. Table 3 summarizes the detailed elemental dopant concentrations of the Al and P ion-implanted 4H-SiC samples (S2 and S3) obtained through SIMS depth profiling.

## 4. Conclusions

We have closely analyzed the structural and surface properties of 4H-SiC single crystals prepared through ion implantation of Al and P. The ion implantation was conducted at room temperature under optimized conditions. Compared to the bulk 4H-SiC single crystals without ion implantation, no significant differences in surface roughness were observed among the ion-implanted samples. Additionally, XRD, TEM, and SAED patterns confirmed that there were no noticeable lattice mismatches in the crystal structure due to ion implantation. This indicates that the ion implantation of Al and P did not induce structural changes in the 4H-SiC single crystals. Therefore, the Al and P ion-implanted 4H-SiC developed in this study is expected to enable accurate quantitative dopant monitoring in SiC power semiconductors without causing changes in the sensitivity of the dopant monitoring equipment. In summary, the ion implantation conditions developed in this study, without the need for post-annealing, are anticipated to be highly effective as reference materials for monitoring n-type and p-type dopant processes in SiC power semiconductor applications.

## Figures and Tables

**Table 1:** Detailed experimental conditions and identification parameters for three different 4H-SiC samples prepared by ion implantation.

Identification	Dopant Elements	Energy (keV)	Dose (Atoms/cm^2^)	Current (μA)	Temperature (°C)	Time (s)
S1	-	-	-		-	-
S2	Al	150	1.00 × 10^14^	3.0	RT	93
S3	P	150	1.00 × 10^14^	3.0	RT	93

**Table 2:** R_a_ and R_rms_ of the three different implanted samples (S1, S2, S3) from the obtained AFM images in Figure 4.

Sample Identification	Dopants	R_a_ (nm)	R_rms_ (nm)
S1	-	0.174	0.218
S2	Al	0.184	0.231
S3	P	0.177	0.223

**Table 3:** Summary of the elemental dopant concentrations for two different ion-implanted 4H-SiC samples (S2 and S3) obtained by SIMS depth profile in Figure 6.

Identification	Dopant	R_p_ (nm)	C_p_ (Atoms/cm^3^)	Dose (Atoms/cm^2^)
S2	Al	179	6.04 × 10^18^	8.31 × 10^13^
S3	P	138	8.92 × 10^18^	1.05 × 10^14^

## Data Availability

The original contributions presented in the study are included in the article, further inquiries can be directed to the corresponding author.
